# ASIC3 Channels Integrate Agmatine and Multiple Inflammatory Signals through the Nonproton Ligand Sensing Domain

**DOI:** 10.1186/1744-8069-6-88

**Published:** 2010-12-08

**Authors:** Wei-Guang Li, Ye Yu, Zhu-Dan Zhang, Hui Cao, Tian-Le Xu

**Affiliations:** 1 Institute of Neuroscience and State Key Laboratory of Neuroscience, Shanghai Institutes for Biological Sciences, Chinese Academy of Sciences, Shanghai 200031, China; 2 Graduate School of Chinese Academy of Sciences, Shanghai 200031, China

## Abstract

**Background:**

Acid-sensing ion channels (ASICs) have long been known to sense extracellular protons and contribute to sensory perception. Peripheral ASIC3 channels represent natural sensors of acidic and inflammatory pain. We recently reported the use of a synthetic compound, 2-guanidine-4-methylquinazoline (GMQ), to identify a novel nonproton sensing domain in the ASIC3 channel, and proposed that, based on its structural similarity with GMQ, the arginine metabolite agmatine (AGM) may be an endogenous nonproton ligand for ASIC3 channels.

**Results:**

Here, we present further evidence for the physiological correlation between AGM and ASIC3. Among arginine metabolites, only AGM and its analog arcaine (ARC) activated ASIC3 channels at neutral pH in a sustained manner similar to GMQ. In addition to the homomeric ASIC3 channels, AGM also activated heteromeric ASIC3 plus ASIC1b channels, extending its potential physiological relevance. Importantly, the process of activation by AGM was highly sensitive to mild acidosis, hyperosmolarity, arachidonic acid (AA), lactic acid and reduced extracellular Ca^2+^. AGM-induced ASIC3 channel activation was not through the chelation of extracellular Ca^2+ ^as occurs with increased lactate, but rather through a direct interaction with the newly identified nonproton ligand sensing domain. Finally, AGM cooperated with the multiple inflammatory signals to cause pain-related behaviors in an ASIC3-dependent manner.

**Conclusions:**

Nonproton ligand sensing domain might represent a novel mechanism for activation or sensitization of ASIC3 channels underlying inflammatory pain-sensing under *in vivo *conditions.

## Background

Acid-sensing ion channels (ASICs) represent a new subgroup of the epithelial sodium channel/degenerin (ENaC/DEG) family of ion channels. To date, functional cloning studies revealed four genes that give rise to at least six ASIC informs (ASIC1a, ASIC1b, ASIC2a, ASIC2b, ASIC3, and ASIC4) [1]. These isoforms, which are composed of cytosolic N and C termini, two transmembrane helices, and a disulfide-rich, multi-domain extracellular region, can associate into homo- or heterotrimers [1,2]. ASICs are amiloride-sensitive voltage-independent cationic channels that are activated by a decrease in extracellular pH [3]. Protons trigger a transient inward current that desensitizes rapidly in all forms of ASICs except ASIC3, which displays a sustained current that does not fully desensitize despite prolonged exposure to acidic extracellular pH [4-7]. ASIC3 is predominantly expressed in sensory neurons and has been shown to be a sensor of acidic and primary inflammatory pain [8,9]. In addition to protons, a synthetic compound, 2-guanidine-4-methylquinazoline (GMQ) has been found to activate ASIC3 channels at physiologically normal pH in a sustained manner [10]. Furthermore, GMQ acts at a site on the ASIC3 that is separate from the known proton binding sites [10]. The identification of this nonproton ligand sensing domain argues that natural ligands beyond protons may activate ASICs under physiological conditions.

Arginine is one of the most versatile amino acids in mammals and has multiple metabolic fates. Not only is it metabolically interconvertible with the amino acids proline and glutamate, but it also serves as a precursor for synthesis of protein, nitric oxide (NO), agmatine (AGM), polyamines, ornithine, and urea [11]. Among these, AGM and polyamines (including spermine, spermidine, and putrescine) are positively charged at physiological pH and thus can interact electrostatically with negatively charged nucleic acids and proteins, including receptors and ion channels. For example, extracellular AGM binds to imidazoline receptors [12,13], and blocks N-methyl-D-aspartate (NMDA) receptors [14] and other ligand- or voltage-gated cation channels [15-17]. However, intracellular spermine and spermidine contribute to the rectification of inward rectifier K^+ ^channels [18,19] and certain types of glutamate receptors [20,21]. Furthermore, polyamines block the Transient Receptor Potential Melastatin (TRPM) channels, TRPM4 [22] and TRPM7 [23], and serve as potent ligands for the capsaicin receptor Transient Receptor Potential Vanilloid Type 1 (TRPV1) [24] and the calcium-sensing receptor [25], a G-protein-coupled receptor that contributes to the regulation of calcium homeostasis. In addition, spermine produces complex effects on NMDA receptors, with either stimulating activity [26,27] or inducing a voltage-dependent blockade [28]. Interestingly, spermine shifts the steady-state desensitization of ASIC1a channels to more acidic pH conditions [29] and contributes significantly to ischemic neuronal injury through enhancing ASIC1a activity [30].

**Figure 1:**
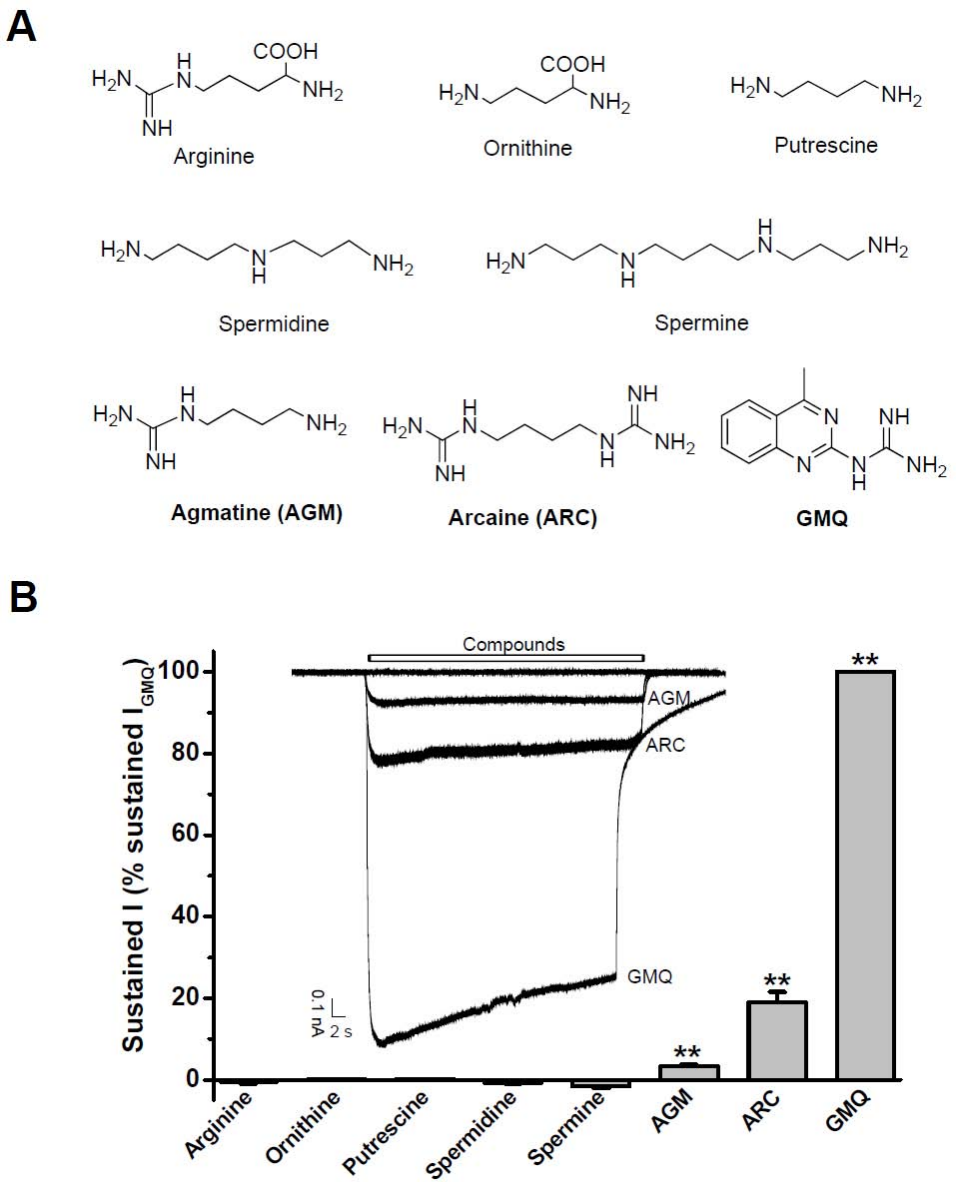
**Effects of Arginine and Its Metabolites on ASIC3 Channels**. (A) Chemical structure of GMQ, arcaine (ARC), arginine and its metabolites. (B) Summary (periphery) of and sample traces (inset) showing the effects of compounds on ASIC3 channels at neutral pH. Each compound was applied at a concentration of 1 mM. The first above trace represents the baseline level without drug application (zero level), followed by the current induced by AGM, ARC, and GMQ, respectively. Data points are means ± S.E.M. of four to six measurements normalized to GMQ-induced response. **p < 0.001 *vs*. baseline level.

Furthermore, peripheral AGM and polyamines are implicated in inflammation and pain signaling. Levels of AGM and polyamines are increased during infection, trauma, and cancer [31,32]. In a previous study, we have shown that extracellular AGM and its structural analog ARC (Figure 1A) activate ASIC3 at neutral pH [10]. In this study, we further showed that AGM-induced activation of ASIC3 is profoundly potentiated by mild acidosis, hyperosmolarity, increased arachidonic acid (AA), or reduced extracellular Ca^2+^, conditions that occur during inflammation and many other pathophysiological processes [8,33-39]. Furthermore, AGM cooperated with the multiple inflammatory signals to cause pain-related behaviors in an ASIC3-dependent manner.

## Results & Discussion

### AGM but Not Polyamines Activates ASIC3 Channels

The fact that AGM directly activates ASIC3 channels [10] prompted us to look for the effects of polyamines and other arginine metabolites [11] (Figure 1A). Conventional whole-cell patch clamp recordings were performed in Chinese Hamster Ovary (CHO) cell lines transiently expressing ASIC3 tagged with GFP to measure the functional activation of ASIC3 channels under voltage clamp conditions. At a concentration of 1 mM, only AGM and its analog ARC (Figure 1B), but not polyamines (including spermine, spermidine, and putrescine), nor L-arginine, nor L-ornithine, were able to activate the ASIC3 channel at pH 7.4. The resistance of ASIC3 to polyamines differs from the modulation of ASIC1a by spermine [29,30] and the modulation of TRPV1 by polyamines [24], arguing for multifunctional roles of arginine metabolites in inflammatory responses [40]. Therefore, as two major sensors for inflammatory pain, ASIC3 and TRPV1 sense different arginine metabolites (AGM of ASIC3 *vs*. polyamines of TRPV1, respectively).

AGM is widely and unevenly distributed in mammalian tissues [41]. Observed first in the rat brain, AGM was shown later that its concentration in the brain (2.40 ng/g) is lower than in others, such as stomach (71.00 ng/g), intestine, spleen and liver (5.63 ng/g) [42]. The relative low concentration of AGM in normal tissues together with the relative low potency of AGM on ASIC3 channels (3.3 ± 0.3% of the GMQ's response, Figure 1B) argue for the negligible contribution of AGM under physiological conditions. However, AGM is detectable in human plasma and higher concentrations have been observed in depressed patients [43]. Similarly, levels of peripheral AGM and polyamines are elevated during infection, trauma, and cancer [31,32]. Interestingly, AGM evokes ASIC3-dependent pain-behavior in mice [10], suggesting that AGM-ASIC3 interaction may become functionally relevant under pathological conditions.

### Synergic Effect of AGM and Mild Acidosis on ASIC3 Channels

**Figure 2:**
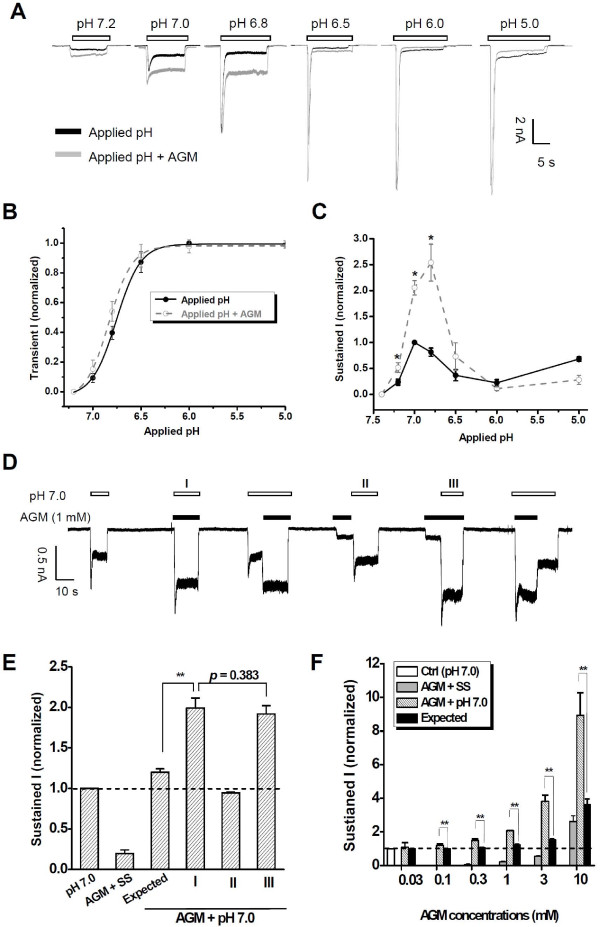
**Mild Acidosis Potentiates AGM-Induced ASIC3 Channel Activation**. (A) Representative traces showing currents induced by acid with graded pH as indicated in the absence (black) or presence (grey) of AGM (1 mM). (B, C) Pooled data as shown in (A) illustrating pH-dependent interaction between AGM- and acid-induced transient (B) or sustained (C) inward currents. Each point is the mean ± S.E.M. of four to five measurements and the solid (black) or dashed (grey) lines are fits to the Hill equation. *p < 0.05, represents the significant difference of current amplitude in the absence or presence of AGM (1 mM). The pH at half maximal activation (pH_50_) values are 6.74 ± 0.02 (*n *= 3.6 ± 0.4) and 6.81 ± 0.02 (*n *= 3.8 ± 0.6) in the absence or presence of AGM, respectively. (D-F) Synergistic interaction between AGM (1 mM) and mild acidosis (pH 7.0). '**I**', '**II**', and '**III**' in (D) indicate co-, pre-, and pre + co-administrations of AGM (1 mM) and mild acid (pH 7.0) as also represented in (E), respectively. The synergistic interaction between AGM and mild acidosis (pH 7.0) is suggested by the two-way ANOVA analysis (*p *< 0.0001). (F) Concentration-dependence of AGM under mild acidosis (pH 7.0). AGM was co-applied as shown in protocol **I **of (D). Data points are means ± S.E.M. of four to five measurements normalized to pH 7.0-induced currents (control, dashed line). Expected value is the linear summation of normalized currents induced by pH 7.0 and AGM individually. **p < 0.001.

Pain conditions are associated with multiple inflammatory signals (mild acidosis, hyperosmolarity, increased arachidonic acid, or reduced extracellular Ca^2+^). To understand the functional relevance of AGM-ASIC3 interaction, we examined the pH dependence of AGM action by applying graded pH to the CHO cell expressing ASIC3 channels, in the presence or absence of AGM (Figure 2A). We found that when the typical biphasic ASIC3 responses were evoked by a pH reduction, AGM dramatically enhanced the sustained component (Figure 2A, C), without altering the peak component (Figure 2A, B). The effect was most evident under mild acidosis conditions (pH 7.2~6.8). This enhancement could be simply the summation of two independent currents induced by AGM and mild acidosis, respectively. To address this issue, we explored the interaction between AGM and pH 7.0 more specifically (Figure 2D-F). We found that pH 7.0 significantly potentiated AGM response and the potentiation was always more than additive with pH reduction regardless the sequence of agonist application (Figure 2D, E), a characteristic that is opposite to the accelerated ASIC desensitization caused by pretreatment with acidic solution [36]. This potentiation occurred only under the simultaneous presence of two ligands (i.e., H^+ ^and AGM) (Figure 2D, E, panels **I **and **III**), suggesting coincident detection of proton and nonproton ligands by ASIC3 channels *in vivo*. Remarkably, a significant potentiation was observed even when AGM was applied at lower concentrations (~100 μM, Figure 2F). The inability of AGM pretreatment to enhance ASIC3 response to mild acidosis (Figure 2D, E, panel **II**) suggests a novel mechanism underlying the observed synergy between H^+ ^and AGM that differs from the allosteric effect of tarantula toxin psalmotoxin 1 (PcTX1) on ASIC1 channels [44,45].

It is well known that peripheral pH falls to < 7 during inflammation, infection, ischemia, hematomas, and exercise [1]. Moreover, such acidosis is well recognized to activate nociceptors and to produce pain in humans that can be attenuated by the ENaC/DEG inhibitor amiloride [46-48]. Additionally, inflammatory mediators, such as nerve growth factor (NGF), serotonin (5-HT), interleukin-1, bradykinin, and brain-derived neurotrophic factor (BDNF) can stimulate ASIC3 transcription, which perhaps contributes to the pain-enhancing effects of these mediators [49,50]. Thus, ASIC3 channels seem to act as a major inflammatory pain integrator [8,9]. Considering that both AGM production [31,32] and ASIC3 expression [51,52] are increased during inflammation, the positive synergy between H^+ ^and AGM in activating ASIC3 channels adds a new level of complexity to the molecular events that can lead to inflammatory pain. The dramatic enhancement under pH 7.2-6.8 (Figure 2C) is reminiscent of a previous observation reporting sustained 'window' current through ASIC3 channels at modest pH changes presumably contributing to myocardial ischemia [36]. Whether AGM regulates cardiac pain-sensing [36] and other forms of muscle pain [9,52,53] awaits further investigations.

### Synergy between AGM and Hyperosmolarity

**Figure 3:**
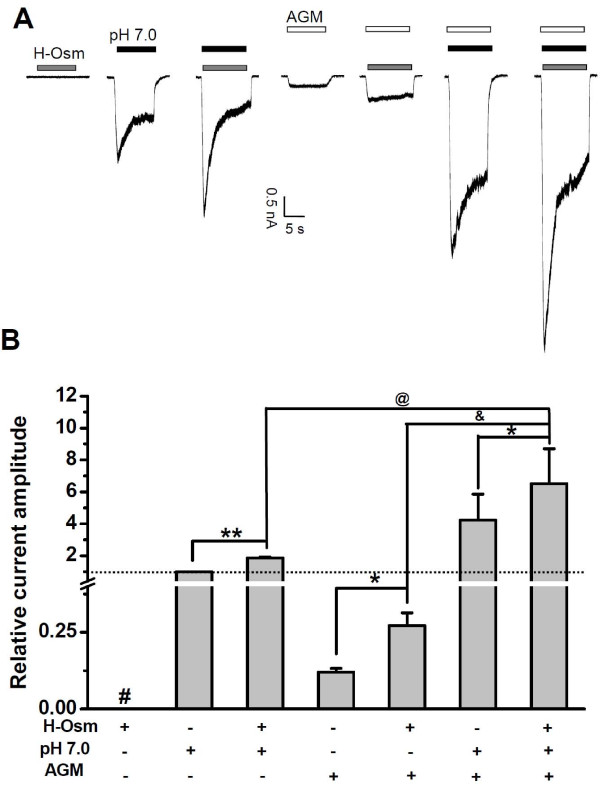
**Hyperosmolarity Potentiates AGM-Evoked ASIC3 Activation**. (A) Typical traces showing a lack of response to hyperosmolarity (600 mosmolkg^-1 ^with mannitol, H-Osm), and currents induced by mild acid (pH 7.0), mild acid with hyperosmolarity (H-Osm + pH 7.0), AGM (1 mM), AGM (1 mM) with hyperosmolarity (H-Osm + AGM), AGM (1 mM) plus mild acid (pH 7.0 + AGM), and AGM (1 mM) with the combined mild acid and hyperosmolarity (H-Osm + pH 7.0 + AGM). (B) Pooled data as shown in (A). Data points are means ± S.E.M. of five to six measurements normalized to pH 7.0-induced current (dashed line). *p < 0.05, **p < 0.001, demonstrate the significant difference of current amplitude in the absence or presence of hyperosmolarity; ^&^*p *< 0.05, demonstrates the significant difference of current amplitude in the absence or presence of mild acid (pH 7.0); ^@^*p *< 0.05, demonstrates the significant difference of current amplitude in the absence or presence of AGM (1 mM).

In inflamed or injured tissues, multiple mediators meet in the interstitial fluid and form an inflammatory exudate, the content of which is acidic [34] and hyperosmotic [37]. Previous studies have shown that hyperosmolarity increases neuronal excitability in DRG neurons [8], affecting preferentially the sustained component of ASIC3 currents. These previous studies promoted us to examine the synergy among hyperosmolarity, acidosis, and AGM. ASIC3-expressing CHO cells were exposed to AGM and mild acidosis in the absence or presence of hyperosmolarity (Figure 3). The hyperosmolarity (600 mosmol kg^-1 ^with mannitol) itself did not induce any detectable current (Figure 3A) but significantly potentiated the ASIC3 currents evoked by a pH reduction to 7.0 (Figure 3), an effect previously observed in rat DRG neurons or an ASIC3 expressing F-11 DRG cell line [8]. Similarly, the current induced by AGM was markedly enhanced by hyperosmolarity (Figure 3). Interestingly, AGM caused further increase over the current induced by the combination of pH 7.0 and hyperosmolarity (Figure 3), suggesting that AGM, mild acidosis, and hyperosmolarity act synergically to facilitate ASIC3 opening, which may explain the enhanced sensory neuronal excitability under conditions of inflammation [8] or cardiac ischemia [36].

### Synergy between AGM and Arachidonic Acid

**Figure 4:**
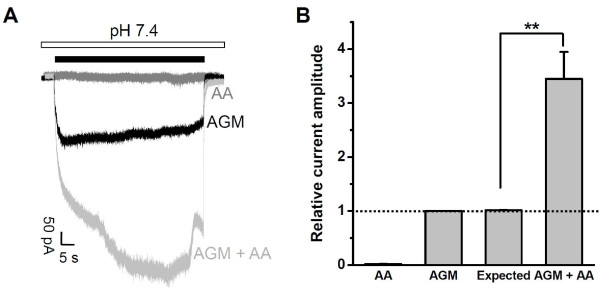
**Arachidonic Acid (AA) Potentiates AGM-Induced ASIC3 Channel Activation**. (A) Representative traces showing currents induced by AA (10 μM, dark grey), AGM (1 mM) in the absence (black) or presence (light grey) of AA (10 μM). (B) Pooled data as shown in (A). Data points are means ± S.E.M. of five measurements normalized to AGM (1 mM)-induced current (dashed line). Expected value is the linear summation of normalized currents induced by AA (10 μM) and AGM (1 mM) individually. **p < 0.001.

Next, we tested arachidonic acid (AA), a pro-inflammatory and ischemic factor which enhances ASIC currents induced by acid and increases neuronal excitability [8,54,55]. At the physiological normal pH, AA induced negligible currents from ASIC3-expressing CHO cells (Figure 4A). As expected, AA significantly potentiated AGM (1 mM)-induced currents (Figure 4). The relatively slow developing kinetics of the AGM current following addition of AA (Figure 4A) presumably reflects the slow onset of AA effect which requires several minutes to be fully established [8]. Therefore, AGM not only activates ASIC3 by itself at normal pH (Figure 1; Ref. [10]), but also exerts positive cooperative effect when administrated with other inflammatory factors such as mild acidosis, hyperosmolarity, and AA, further strengthening the notion that ASIC3 channels act as a multiple sensor to integrate diverse signals present in the pathophysiological environment [8,9].

### AGM, Ca^2+^, and ASIC3 Channel Activation

**Figure 5:**
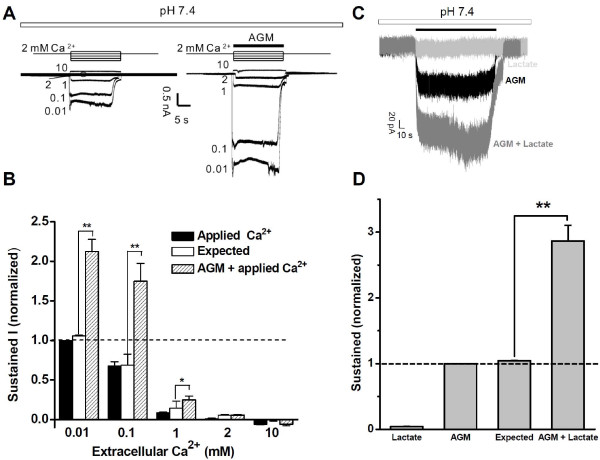
**Reduced Extracellular Ca^2+ ^Potentiates AGM-Induced ASIC3 Channel Activation**. (A) Representative traces showing currents induced by reduced extracellular Ca^2+ ^(left) and 1 mM AGM applied under low Ca^2+ ^(right). (B) Pooled data as shown in (A). Data points are means ± S.E.M. of four to five measurements normalized to currents evoked with 0.01 mM Ca^2+ ^(dashed line). Expected value is the linear summation of normalized currents induced by reduced Ca^2+ ^and 1 mM AGM under low Ca^2+ ^conditions individually. *p < 0.05, **p < 0.001. The two-way ANOVA analysis suggests the synergetic interaction between AGM and Ca^2+ ^reduction (*p *< 0.01). (C) Representative traces showing currents induced by lactate (15 mM, keeping the pH neutral, light grey), AGM (1 mM) in the absence (black) or presence (dark grey) of 15 mM lactate. (D) Pooled data as shown in (C). Data points are means ± S.E.M. of five measurements normalized to AGM (1 mM)-induced currents (dashed line). Expected value is the linear summation of normalized currents induced by lactate (15 mM) and AGM (1 mM) individually. **p < 0.001.

We also investigated the sensitivity of AGM-induced currents to alterations of extracellular Ca^2+^, which has marked effect on the GMQ response [10]. As shown in Figure 5A, B, the presence of 10 mM extracellular Ca^2+ ^completely abolished the AGM currents, whereas reducing Ca^2+ ^significantly potentiated AGM-induced currents. Low Ca^2+ ^itself evoked a significant inward current (Figure 5A, left panel), consistent with a previous report [56]. Thus, similar to GMQ [10], AGM-ASIC3 interaction is highly sensitive to altered extracellular Ca^2+^. The extracellular Ca^2+ ^concentration can decrease from a resting value of around 1.2-1.8 mM to values as low as 0.08 mM under certain conditions [38], suggesting that the signaling cascade induced by AGM-ASIC3 interaction might be markedly amplified under such conditions. Moreover, lactate produced by anaerobic metabolism reduces extracellular Ca^2+ ^concentration and results in the enhancement of the acid-induced ASIC currents in ischemia-sensing neurons [39]. Likewise, AGM-evoked currents were increased about 3-folds when lactate and AGM were co-applied (Figure 5C, D). These results suggest that ASIC3 channels may be gated *in vivo *by the combined actions of reduced extracellular Ca^2+^, mild acidosis, and AGM through synergic interactions reported here.

It is, however, possible that AGM-induced ASIC3 channel activation was through the chelation of extracellular Ca^2+ ^as observed with lactate [39,56] at neutral pH. To clarify this possibility, we recorded ASIC3 response to AGM in Ca^2+^-free external solution. We found that AGM activated ASIC3 channels regardless the presence or absence of extracellular Ca^2+ ^(data not shown). That AGM activates ASIC3 channels independent of Ca^2+ ^chelation is consistent with the notion that AGM modulates ASIC3 activation via novel mechanisms (Figure 2D, E).

### Critical Role of the Nonproton Ligand Sensing Domain

**Figure 6:**
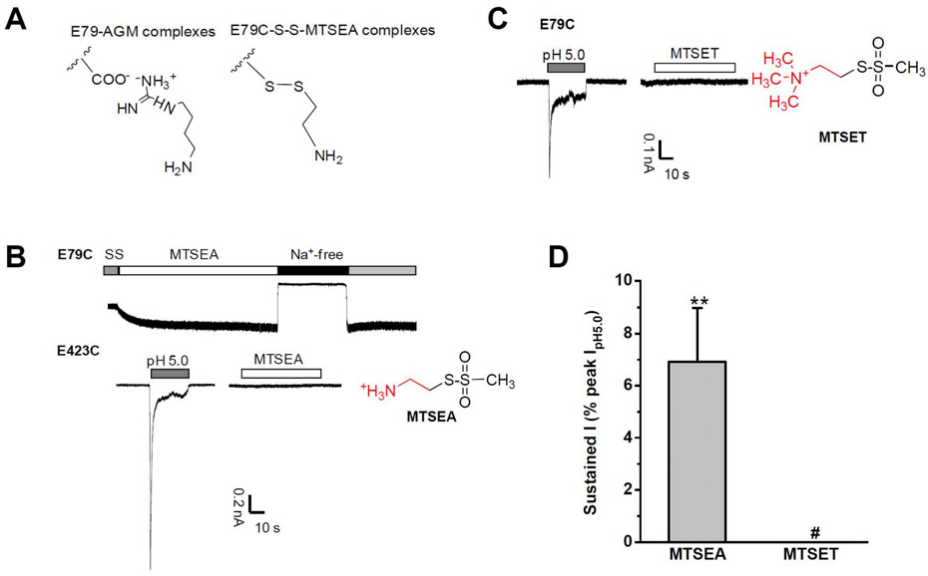
**Effects of MTS Reagents on ASIC3^E79C ^Channels at Neutral pH**. (A) Models showing the structural similarity between MTSEA and AGM in activating ASIC3. (B) Sample traces showing effects of MTSEA (0.2 mM) on ASIC3^E79C ^(upper trace) and ASIC3^E423C ^mutated channels (lower traces) at pH 7.4. Similar results were obtained from other four measurements. (C) Sample traces showing a lack of effects of MTSET (0.5 mM) on ASIC3^E79C^. (D) Maximal currents induced in ASIC3^E79C ^by MTSEA (0.2 mM, 2 min) and MTSET (0.5 mM, 5-10 min). Data points are means ± S.E.M. of five measurements normalized to pH 5.0-induced peak currents. **p < 0.001 *vs*. baseline level. ^#^indicates no detectable changes upon exposure to MTSET (0.5 mM, 5-10 min).

Next we asked how AGM activates ASIC3 channels in a manner similar to GMQ, given the difference in their structural flexibility (linear AGM *vs*. circular GMQ with a heterocycle, Figure 1A) [10]. In a previous study, we have shown that the nonproton ligand sensing domain plays a critical role in mediating AGM and GMQ effects on ASIC3 [10]. While its critical role for GMQ was supported by the fact that covalently linking circular GMQ or TNB to C79 activated ASIC3^E79C ^channels (with the GMQ-dimer or DTNB treatment) [10], the role of the same site for the linear AGM was not established. For comparison, we tested 2-aminoethyl-methanethiosulfonate (MTSEA), a linear thiol-reactive compound on ASIC3^E79C ^channels, in which the residue Glu79 was replaced by a cysteine, thus mimicking AGM-E79 interaction (Figure 6A). Bath application of 0.2 mM MTSEA persistently activated ASIC3^E79C ^channels at pH 7.4 (Figure 6B, D). By contrast, MTSEA (0.2 mM) was ineffective in CHO cells expressing wild-type (WT, data not shown) or ASIC3^E423C ^channels (Figure 6B). Interestingly, 2-(trimethylammonium)ethyl methanethiosulfonate (MTSET, 0.5 mM), a MTS reagent in which the amino group is replaced by a trimethylamine, failed to induce any detectable currents in ASIC3^E79C^-expressing CHO cells (Figure 6C), suggesting that the amino group in AGM plays an essential role in activating ASIC3 channels, as has been shown in GMQ-ASIC3 interaction [10]. Together, these data strongly support that activation of ASIC3 by AGM relies on polar, steric, and electrostatic interactions with the nonproton ligand sensing domain in the channel [10], regardless whether the ligand is linear and flexible (i.e. AGM) or circular and rigid (i.e. GMQ).

### ASIC Subunit Specificity

**Figure 7:**
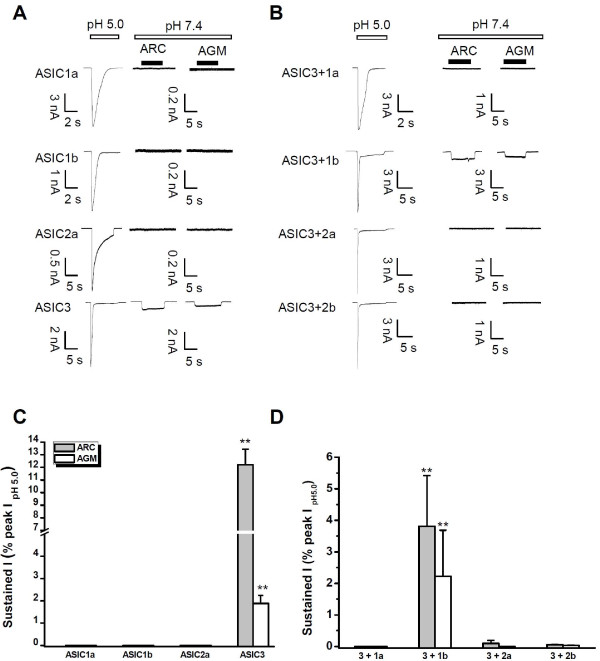
**Subunit Specificity of Agmatine (AGM) and Arcaine (ARC) in CHO Cells Transfected with One or Two Different ASIC Subunits**. (A, B) Representative traces showing the response of AGM (1 mM) or ARC (1 mM) on CHO cells transfected with one (A) or two (B) different ASIC subunits. (C, D) Polled data as shown in (A, B). AGM (1 mM) and ARC (1 mM) directly activate ASIC3 homomeric channels (A, C) and heteromeric ASIC3 + 1b channels (B, D) but not ASIC1a nor ASIC1b nor ASIC2a homomeric channels (A, C), nor heteromeric ASIC3 + 1a, ASIC3 + 2a, ASIC3 + 2b channels (B, D) at the neutral pH (pH = 7.4). Data are means ± S.E.M. n = 3-5. **p < 0.001 *vs*. baseline level.

Most ASIC-like acid-evoked currents in DRG neurons are mediated by heteromers of ASIC3, -2, and -1 [57]. To address ASIC subunit specificity, we recorded AGM or ARC responses in CHO cells expressing different combinations of ASIC subunits (Figure 7). Similar to GMQ [10], none of the homomeric channels ASIC1a, ASIC1b, or ASIC2a were activated by either AGM or ARC (Figure 7A, C). However, heteromeric ASIC3 plus ASIC1b channels responded to AGM and ARC (Figure 7B, D) in a manner similar to homomeric ASIC3 channels (Figure 7A, C). On the other hand, heteromeric combinations of ASIC3 plus ASIC1a, ASIC2a, or ASIC2b were insensitive to these ligands (Figure 7B, D). This subunit specificity together with the restricted distribution pattern of ASIC3 [9] suggests that an AGM-dependent regulatory pathway most likely occurs in peripheral tissues expressing homomeric ASIC3 and/or heteromeric ASIC3 + 1b channels [8,36,58]. Alternatively, AGM may act as a co-agonist sensitizing the apparently-unresponsive heteromeric ASIC3 channels (i.e., ASIC3 + 1a, 2a, or 2b) (Figure 7) to protons. In addition, according to a recent report [59], AGM may also act on the ASIC3 channels through coincident detection of multiple ligands (i.e., AGM, H^+^, and other factors such as ATP) by cross-activating an as yet unidentified ion channel. In addition, the emergence of new ASIC isoforms [60] adds an additional possibility underlying the ASIC3-dependent pain-behavior induced by AGM *in vivo *[10].

### ASIC3 Channels Integrate Multiple Inflammatory Signals *in vivo*

**Figure 8:**
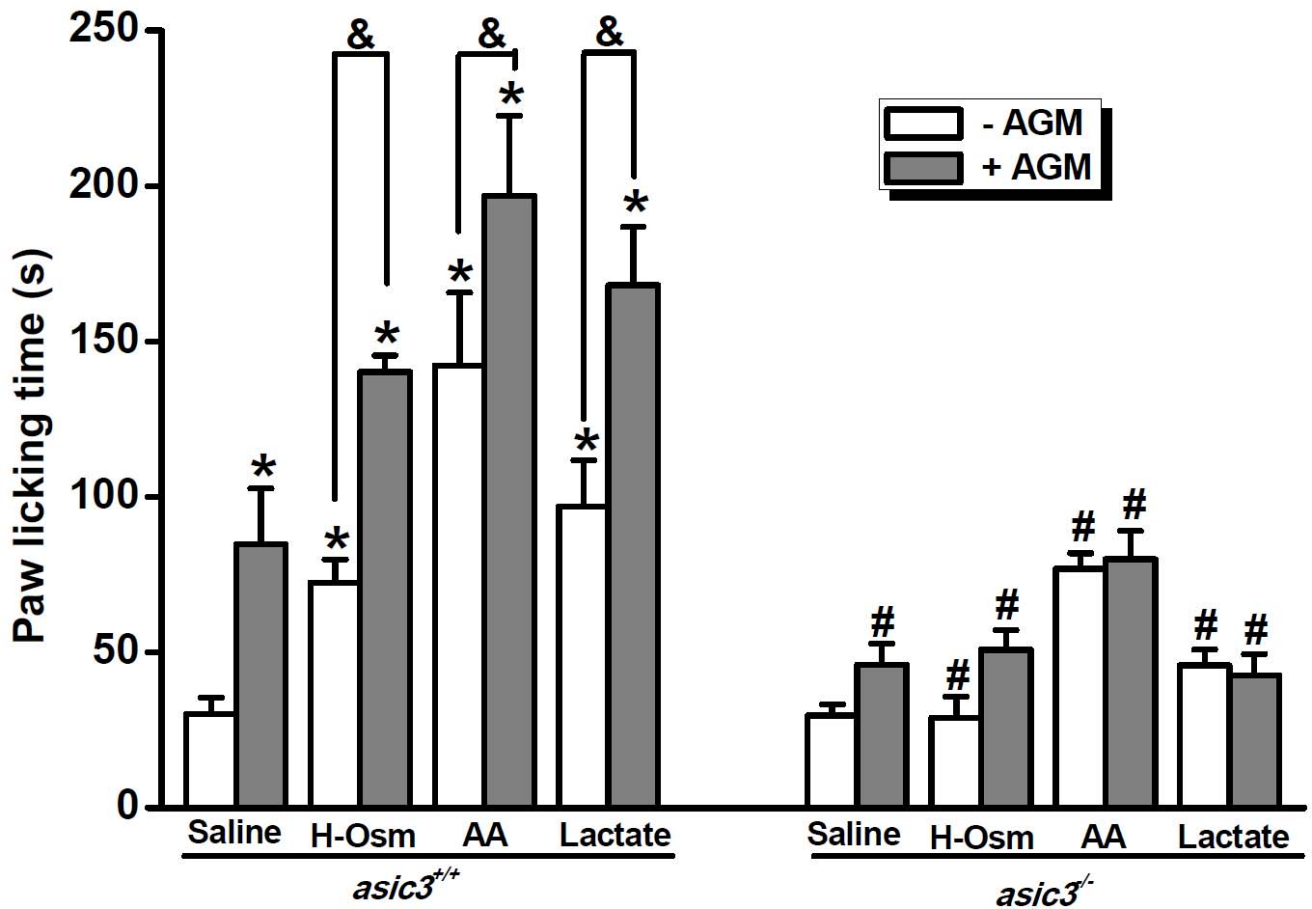
**AGM Causes Pain-Related Behaviors via ASIC3 Channels**. Pain-related behavior as determined by the time spent for paw licking following saline or AGM (1 mM) injection (10 μl) in the absence (white) or presence (grey) of hyperosmolarity (H-Osm, 600 mosmolkg^-1 ^with mannitol), AA (10 μM), or lactate (15 mM, keeping the pH neutral). Data are means ± S.E.M. n = 6-9. *p < 0.05 versus saline; ^#^*p *< 0.05, *asic3*^-/- ^versus *asic3*^+/+^; ^&^*p *< 0.05.

Finally, to gain insights into the pathophysiological relevance of ASIC3-dependent integration of AGM and multiple inflammatory signals, we performed *in vivo *pain-related behavioral tests [10] following the injection of AGM and hyperosmolarity, arachidonic acid (AA), or lactate into the right hindpaw of *asic3*^+/+ ^and *asic3*^-/- ^mice. We measured the total time the animals spent licking the injected paw during a 30-min period. As shown previously, control *asic3^+/+ ^*mice showed a significant increase in paw-licking time after AGM (10 mM) injection compared to saline-injected controls [10]. In consideration of the high AGM concentration used, we re-evaluated the paradigms by injecting 1 mM AGM (Figure 8). Similarly, *asic3^+/+ ^*mice showed a significant increase in paw-licking time after AGM (1 mM) injection, though less intense than that observed following 10 mM AGM injection [10]. As expected, the reaction of *asic3*^-/- ^mice to AGM was significantly reduced (Figure 8). When AGM (1 mM) was co-applied with hyperosmolarity (H-Osm, 600 mosmol kg^-1 ^with mannitol), AA (10 μM), or lactate (15 mM, keeping the pH neutral), the injection elicited more intense response in *asic3^+/+ ^*mice while failed to elicit the comparable response in *asic3^-/- ^*mice. Interestingly, *asic3^+/+ ^*mice showed a significant increase in paw-licking time following the treatment of H-Osm (600 mosmol kg^-1 ^with mannitol), AA (10 μM), or lactate (15 mM) alone compared to saline-injected controls. These behavioral responses were similarly reduced in *asic3^-/- ^*mice (Figure 8), supporting an essential role of ASIC3 in sensing multiple inflammatory signals [9], including AGM.

## Conclusions

ASIC3 channels sense extracellular protons and nonproton ligands, including the endogenous molecule AGM, which is a metabolite of arginine. In this study, we extended the previous finding that ASIC3 can be activated by small molecules with basic groups such as GMQ, AGM, and ARC by uncovering the functional interactions of AGM with multiple inflammatory factors such as hyperosmolarity, arachidonic acid, and lactate. Cysteine modification with a linear thiol-reactive compound that mimics AGM binding induces ASIC3 opening in a sustained manner similar to AGM, supporting the critical role of the newly identified nonproton ligand sensing domain. *In vivo *tests using both *asic3^+/+ ^*and *asic3^-/- ^*mice revealed that AGM cooperates with the multiple inflammatory signals to cause pain-related behaviors in an ASIC3-dependent manner. Thus, the present findings suggest a new mechanism for activation or sensitization of ASIC3 channels underlying inflammatory pain-sensing under *in vivo *conditions.

## Methods

### Cell Culture and Transfection

All constructs were expressed in CHO cells as described previously [10]. In brief, CHO cells were cultured at 37 °C in a humidified atmosphere of 5% CO_2 _and 95% air. The cells were maintained in F12 medium (INVITROGEN) supplemented with 1 mM L-glutamine, 10% fetal bovine serum, 50 units/ml penicillin, and 50 μg/ml streptomycin. Transient transfection of CHO cells was carried out using Lipofectamine™2000 (INVITROGEN). Electrophysiological measurements were performed 24-48 h after transfection.

### Solutions and Drugs

The ionic composition of the incubation solution (SS, see Figures 2E, F and 6B) was (mM): 150 NaCl, 5 KCl, 1 MgCl_2_, 2 CaCl_2_, 10 HEPES, and 10 glucose, aerated with 95% O_2_/5% CO_2 _to a final pH of 7.4. The standard external solution contained (mM): 150 NaCl, 5 KCl, 1 MgCl_2_, 2 CaCl_2_, and 10 glucose, buffered to various pH values with either 10 mM HEPES, pH 6.0-7.4, or 10 mM MES, pH < 6.0. For the Na^+^-free medium (Figure 6B), Na^+ ^was substituted with equimolar *N*-Methyl-D-glucamine (NMDG). The patch pipette internal solution for whole-cell patch recording was (mM): 120 KCl, 30 NaCl, 1 MgCl_2_, 0.5 CaCl_2_, 5 EGTA, 2 Mg-ATP, and 10 HEPES. The internal solution was adjusted to pH 7.2 with Tris-base. The osmolarities of all these solutions were maintained at 300-325 mOsm (Advanced Instrument, Norwood, MA). Hyperosmotic (H-Osm) conditions were obtained by adding mannitol to the standard external solution (or saline) as indicating in the text.

Solutions with different composition were applied using a rapid application technique termed the "Y-tube" method throughout the experiments [10]. This system allows a complete exchange of external solution surrounding a cell within 20 ms.

### Site-Directed Mutagenesis

The cDNA of rat ASIC3 was subcloned into the pEGFPC3 vector (Promega Corporation, Madison, WI, U.S.A.). Each mutant was generated with the QuikChange^® ^mutagenesis kit (Stratagene, La Jolla, CA) in accordance with the manufacturer's protocol using high-performance-liquid-chromatography-purified or PAGE-purified oligonucleotide primers (Sigma-Genosys, The Woodlands, TX). Individual mutations were verified by DNA sequence analysis, and the predicted amino acid sequences were determined by computer analysis.

### Electrophysiology

The electrophysiological recordings were performed using the conventional whole-cell patch recording configuration under voltage clamp condition. Patch pipettes were pulled from glass capillaries with an outer diameter of 1.5 mm on a two-stage puller (PP-830, Narishige Co., Ltd., Tokyo, Japan). The resistance between the recording electrode filled with pipette solution and the reference electrode was 3-5 MΩ. Membrane currents were measured using a patch clamp amplifier (Axon 700A, Axon Instruments, Foster City, CA) and were sampled and analyzed using a Digidata 1320A interface and a computer running the Clampex and Clampfit software (version 8.0.1, Axon Instruments). In most experiments, 70-90% of the series resistance was compensated. Unless otherwise noted, the membrane potential was held at -60 mV throughout the experiment under voltage clamp conditions. All the experiments were carried out at room temperature (23 ± 2 °C).

### Pain-Related Behavioral Assays

Animals were acclimatized for 30 min before experiments. A total volume of 10 μl solution (in 0.9% NaCl) containing either saline (0.9% NaCl only), or AGM (1 mM), or hyperosmolarity (H-Osm, 600 mosmol kg^-1 ^with mannitol), or AA (10 μM), or lactate (15 mM), or AGM + H-Osm, or AGM + AA, or AGM + lactate was injected intraplantarly using a 30G needle and paw-licking behavior was quantified for 30 min [10].

### Data Analysis

Results were expressed as means ± S.E.M. Unless otherwise noted, statistical comparisons were made with the Student's *t *test.*, or ^&^, or ^@^, *p *< 0.05 or **p < 0.001 was considered significantly different. To test the synergic interaction between two factors (i.e., mild acidosis and AGM, or AGM and Ca^2+ ^reduction) on ASIC3 currents, additional two-way ANOVA analyses were made (Figures 2 and 5, *p *< 0.05 was considered significant). Concentration-response relationships for pH-dependent activation of ASIC3 channels were obtained by measuring currents in response to acidic solutions with graded pH values. Each acidic solution was tested on at least three CHO cells and all results used to generate a concentration-response relationship were from the same group. The data were fit to the Hill equation: *I*/*I*_max _=1/[1+(*EC*_50_/[*Ligand*])^*n*^], where *I *is the normalized current at a given pH, *I*_max _is the maximum normalized current, *EC*_50 _is the concentration of proton yielding a current that is half of the maximum, and *n *is the Hill coefficient.

## Abbreviations

5-HT: serotonin; AA: arachidonic acid; AGM: agmatine; ARC: arcaine; ASIC: acid-sensing ion channel; BDNF: brain-derived neurotrophic factor; CHO: Chinese Hamster Ovary; DRG: dorsal root ganglion; ENaC/DEG: epithelial sodium channel/degenerin; GFP: green fluorescent protein; GMQ: 2-guanidine-4-methylquinazoline; H-Osm: Hyperosmolarity; MTSEA: 2-aminoethyl-methanethiosulfonate; MTSET: 2-(trimethylammonium)ethyl methanethiosulfonate; NGF: nerve growth factor; NMDA: *N*-methyl-D-aspartate; NO: nitric oxide; PcTX1: psalmotoxin 1; TRPM: Transient Receptor Potential Melastatin; TRPV1: Transient Receptor Potential Vanilloid Type 1; WT: wild-type.

## Competing interests

The authors declare that they have no competing interests.

## Authors' contributions

WGL, YY, and TLX designed the project. WGL, YY, and ZDZ performed cell culture, patch-clamp recording, behavior tests, and data analysis. ZDZ and HC did mutations. WGL and TLX wrote the manuscript. All authors read and approved the final manuscript.
